# Investigation of Ki67 and Phospho-Histone H3 Expressions in Urothelial Carcinoma of the Bladder by Immunohistochemical Method

**DOI:** 10.7759/cureus.55297

**Published:** 2024-02-29

**Authors:** Ramazan Oğuz Yüceer, Şirin Başpınar

**Affiliations:** 1 Medical Pathology, Batman Training and Research Hospital, Batman, TUR; 2 Medical Pathology, Süleyman Demirel University Faculty of Medicine, Isparta, TUR

**Keywords:** digital pathology, phh3, ki67, urothelial carcinoma, bladder

## Abstract

Background: In our study, it is aimed to investigate the relationship between Ki67 and phospho-histone H3 (pHH3) expressions in bladder urothelial carcinomas, with clinicopathological parameters and survival, which have prognostic value.

Methods: The study included 44 cases of high-grade urothelial carcinoma (HGUC), 37 cases of low-grade urothelial carcinoma (LGUC), and 11 nontumoral bladder cases. Ki67 and pHH3 were applied to the paraffin blocks of the tissues of 81 urothelial carcinoma and 11 nontumoral bladder cases by immunohistochemical method. Percentages of Ki67 and pHH3 expressions were evaluated by digital imaging analysis method. Expression percentages were compared with various clinicopathological parameters, and the relationship between them was evaluated.

Results: Ki67 was expressed in 28% of urothelial carcinoma cases and 1% of nontumoral cases. pHH3 was expressed in 10.32% of urothelial carcinoma cases and 0.16% of nontumoral cases. In our study, we found significantly higher Ki67 and pHH3 expressions in urothelial carcinoma compared to nontumoral cases. There was a statistically significant relationship (p < 0.05) and a positive correlation between Ki67 expression and lymphovascular invasion, pT stage, and histological grade. A statistically significant relationship (p < 0.05) and a positive correlation were found between pHH3 expression and lymphovascular invasion, pT stage, recurrence, and histological grade. In addition, a statistically significant relationship was found between Ki67 and pHH3 expressions. In our study, survival was found to be low in high-grade urothelial carcinoma cases with lymphovascular invasion, advanced age (65 years and older), and high Ki67 and pHH3 expression rates.

Conclusions: According to our findings, high Ki67 and pHH3 expressions were found to be associated with poor prognostic parameters such as advanced pathologic stage, high histologic grade, and low survival. Our findings suggest that Ki67 and pHH3 may play a role in the differentiation, progression, and aggressive behavior of urothelial carcinoma. However, further studies are needed to confirm our findings and determine the role of these markers in urothelial carcinoma.

## Introduction

Bladder cancer is the second most common genitourinary system cancer after prostate cancer [1]. Approximately 90% of bladder cancers are urothelial carcinomas. About 70%-80% of bladder cancers are non-muscle invasive (Stage Ta, Tis, T1), representing superficial urothelial carcinomas, while 20%-30% are infiltrative tumors that invade muscle, perivesical soft tissue, and other organs (Stage T2, T3, T4). The five-year survival rate is over 90% for superficial bladder carcinomas, but it has been reported to range between 45% and 55% for muscle tissue-invasive bladder carcinomas [2]. Research on the carcinogenesis of bladder cancer, a prevalent tumor, and advancements in diagnostic and treatment methods are ongoing [3]. In bladder cancer, key prognostic indicators include the patient's age, tumor histological type, tumor stage, and the presence of lymphovascular invasion [4]. Additionally, independent studies have demonstrated that immunohistochemical Ki67 expression is a significant factor [5].

Ki67 is a nonhistone nuclear protein expressed by cells in the proliferative G1, G2, M, and S phases of the cell cycle, but it is not expressed in the G0 phase [5]. The immunohistochemical evaluation of monoclonal antibodies developed against the Ki67 antigen, such as MIB-1 (the most commonly used), is closely associated with tumor aggressiveness and prognosis in numerous cancer types [6]. Studies on Ki67 in bladder urothelial carcinoma have highlighted that high Ki67 expression is an independent risk factor for prognosis and is linked to a poor prognosis [6]. While Ki67 appears to be one of the most promising biomarkers in bladder urothelial carcinomas today, it is noted that further research is required to support this perspective [7].

Phospho-histone H3 (pHH3) protein is a core protein present in the nuclear chromatin of cells, which was discovered in recent years. While it does not affect the interphase period of mitosis in mammalian cells, it plays a significant role in chromatin condensation [8]. In contrast to Ki67, which is expressed in the G1, S, G2, and M phases of mitosis, pHH3 is exclusively expressed during the late G2 phase. Therefore, pHH3 is highly specific for mitosis and is more suitable as a mitosis marker [8,9].

Numerous studies in the literature have explored the expressions of both Ki67 and pHH3 in various tumors, including breast, brain, endometrium, prostate, ovarian, colorectal, head and neck cancers, as well as lymphoma and melanoma. These studies have demonstrated that elevated expression of these two markers is associated with poor prognosis and reduced survival in these tumors [10-16].

Today, histological tumor grade and stage are the most important parameters used in patient treatment and prognosis in urothelial tumors of the bladder. Furthermore, ongoing research is dedicated to exploring markers that might aid in discerning the tumor's biological characteristics. A review of the literature reveals that there have been very few investigations into the combined immunohistochemical expressions of these two markers in bladder cancer and their potential prognostic significance [16-18]. These studies have highlighted that the immunohistochemical expressions of Ki-67 and pHH3 are associated with advanced tumor stage, reduced survival rates, and an unfavorable prognosis.

In our study, our objective was to explore the immunohistochemical expressions of Ki67 and pHH3 in bladder urothelial carcinomas through the utilization of image analysis methods. We aimed to assess the associations between the expressions of these markers and the clinicopathological characteristics of the cases and to investigate the implications of their integration into routine clinical practice.

## Materials and methods

Case selection

The study included 44 cases of high-grade urothelial carcinoma (HGUC), 37 cases of low-grade urothelial carcinoma (LGUC), and 11 nontumoral bladder cases. These cases were diagnosed between 2013 and 2019 at Süleyman Demirel University Faculty of Medicine, Department of Medical Pathology, and were obtained by retrospectively reviewing the hospital's electronic data. Among the cases diagnosed with HGUC and LGUC based on bladder transurethral resection (TUR) materials, the study included patients who were newly diagnosed with urothelial carcinoma, did not have variant histology, and had not received any prior medical treatment for this condition. Hematoxylin-Eosin (H&E)-stained sections of all cases archived in the Department of Medical Pathology were re-evaluated to determine invasion status according to the World Health Organization (WHO) 2016 classification and pathological tumor (pT) staging based on the tumor node metastasis (TNM) system.

For the control group, TUR materials containing nonneoplastic bladder tissue were utilized. The areas that most accurately represented the tumor were identified on H&E sections, and paraffin blocks containing these tissues were selected and retrieved from the archive.

Immunohistochemistry

For the immunohistochemical examination, paraffin blocks of the H&E-stained sections that provided the best representation of the tumor, with minimal areas of necrosis and bleeding, and showed the presence of the lamina propria and muscularis propria were chosen. The sections with a thickness of 4 μm of paraffin blocks were mounted on positively charged slides, with one section for each of the two different tissues. The tissue samples on lysine-coated slides were subjected to deparaffinization by being placed in an oven at 60°C for 8 hours.

Slides were tested with Ki-67 (Agilent, 60-minute incubation, MIB-1 clone, 1/100 concentration) and pHH3 (Agilent, 60-minute incubation, Polyclonal, 1/120 concentration) antibodies. The staining process was carried out on the Dako-Omnis fully automated staining device, utilizing Agilent primary antibodies and kits. Tonsil tissue served as a positive external control for both Ki-67 and pHH3.

Immunohistochemical evaluation

All slide images were digitally evaluated by scanning the Ki67 and pHH3 stained slides under a "Nikon Eclipse Ni-U" light microscope from the hot spot areas under a 10x and 20x objective, using the "Microvisioneer" manual whole slide scanner equipment and its associated Microvisioneer software.

Analysis of digital data

Slides containing both normal and neoplastic bladder tissues were scanned using the "QuPath" open-source digital pathology software. Annotations, which included drawings on the slides, and 3D digitized graphic data were generated from these digitalized slides. The image analysis program was then used to assess the total number and percentage of cells expressing the Ki67 and pHH3 markers through immunohistochemical methods in both normal and neoplastic bladder tissues.

Statistical analysis

Statistical analyses were conducted using SPSS (Statistical Package for the Social Sciences) software, version 26.0 (IBM Corp., Armonk, NY). The normal distribution suitability of the variables was assessed through visual and analytical methods, including the Kolmogorov-Smirnov and Shapiro-Wilk tests. Descriptive statistics were presented as numbers, percentages, means, and standard deviations.

To compare the nontumoral urothelium and urothelial carcinoma groups, a student's t-test was employed. Mann-Whitney U, Pearson Chi-square, Kruskal-Wallis, and post hoc tests were utilized to evaluate the relationships between clinicopathological data of tumor cases and the expressions of Ki67 and pHH3. The correlation between variables was assessed through Spearman correlation analysis. For survival analysis, Kaplan-Meier analysis tests were employed. Statistical significance was considered when the p-value was below 0.05.

Ethics committee approval

The study received the required approval from the Süleyman Demirel University Faculty of Medicine Clinical Research Ethics Committee on November 5, 2021, with report number 311.

## Results

Our study included 81 cases of urothelial carcinoma and 11 nontumoral bladder cases. The average age of the nontumoral cases was 58.45 ± 13.4 years (33-79 years). Among the nontumoral cases, eight (73%) were males, and three (27%) were females. The average age of the urothelial carcinoma cases was 67.54 ± 13.1 years (21-99 years). Among these cases, 68 (84%) were male, and 13 (16%) were female. The clinicopathological findings of the cases are summarized in Table 1.

**Table 1:** Clinicopathological features of urothelial carcinoma cases LGUC: Low-grade urothelial carcinoma; HGUC: High-grade urothelial carcinoma.

Category	(n%)
Age (year)	
<65	36 (44.4%)
≥65	45 (55.6%)
Gender	
Male	68 (84%)
Female	13 (16%)
Relapse	
Yes	15 (18.5%)
No	66 (81.5%)
Histological grade	
LGUC	37 (45.7%)
HGUC	44 (54.3%)
Pathologic tumor stage	
pTa	33 (35.9%)
pT1	30 (32.6%)
pT2	18 (19.6%)
Lymphovascular invasion	
Yes	5 (6.2%)
No	76 (93.8%)
Smoking	
Yes	22 (27.1%)
No	59 (62.9%)
Death	
Yes	38 (46.9%)
No	43 (53.1%)

**Figure 1:**
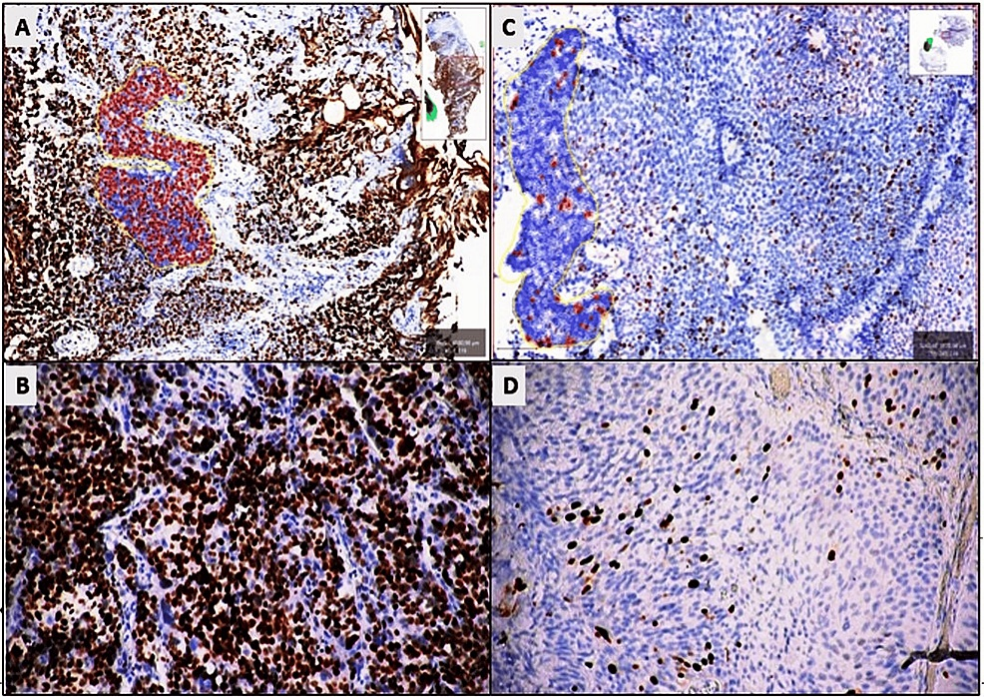
The average Ki67 expression of urothelial carcinoma (A, B) Nuclear expression in 86.6% of cells with Ki67 in muscle-invasive HGUC (QuPath 3.0, DAB x200). (C, D) Nuclear expression with Ki67 in 4.2% of cells in noninvasive LGUC (QuPath 3.0, DAB x200). HGUC: High-grade urothelial carcinoma; LGUC: Low-grade urothelial carcinoma; pHH3: Phospho-histone 3.

In nontumoral bladder tissues, Ki67 expression was limited to a few basal cells in the urothelium, whereas nuclear staining was observed in varying proportions in tumor cells of urothelial carcinoma (Figure 1, Panels A-D). The average number of cells expressing Ki67 in the hot spot area of the tumor was 2313.9 ± 1209, with an average Ki67 expression percentage of 28.0 ± 23.2, ranging from 0.86 to 86.6. In nontumoral urothelium, the average number of cells expressing Ki67 in the hot spot area was 190.0 ± 303.4, and the average Ki67 expression percentage was 1.0 ± 1.9, ranging from 0.0 to 6.58 (Figure 1).

A statistically significant relationship was observed between the mean percentage of Ki67 expression in the urothelial carcinoma and nontumoral bladder groups (p < 0.0001, student's t-test). When examining the relationship between Ki67 expression and pT stage, the average Ki67 expression rates were 9.16 ± 8.32 in pTa, 31.54 ± 17.53 in pT1, and 56.62 ± 17.54 in pT2. A statistically significant relationship was detected between Ki67 expression and the pT stage (p < 0.0001, student's t-test).

The relationship between Ki67 expression and clinicopathological parameters is summarized in Table 2. A statistically significant relationship was detected between Ki67 expression and lymphovascular invasion, pT stage, histological grade, and the presence of death (p < 0.05, Pearson Chi-square test). Additionally, a positive correlation was observed between the Ki67 expression percentage and lymphovascular invasion (p = 0.011, r = 0.281), pT stage (p < 0.001, r = 0.803), and histological grade (p < 0.001, r = 0.822, Spearman correlation test). No statistically significant relationship was observed between Ki67 expression and age, gender, the presence of recurrence, and smoking (p > 0.05), as shown in Table 2.

**Table 2:** Relationship between Ki67 and pHH3 expressions of clinicopathological parameters * Pearson Chi-Square test. ** Post hoc tests. LGUC: Low-grade urothelial carcinoma; HGUC: High-grade urothelial carcinoma; pHH3: Phospho-histone 3.

Category	Ki67 Percentage Expression, Mean ± SD	p-value	pHH3 Percentage Expression, Mean ± SD	p-value
Age (year)				
<65	26.18 ± 23.70	0.529	11.09 ± 13.92	0.657*
≥65	29.45 ± 23.02		9.71 ± 13.95	
Pathologic tumor stage				
pTa	9.16 ± 8.32		3.45 ± 4.12	
pT1	31.54 ± 17.53	<0.0001	9.35 ± 9.70	<0.0001**
pT2	56.62 ± 17.54		24.54 ± 20.13	
Histologic grade				
LGUC	8.51 ± 8.09	<0.0001	3.22 ± 2.95	<0.0001*
HGUC	44.38 ± 19.52		16.21 ± 16.52	
Gender				
Male	27.94 ± 23.17	0.962	10.22 ± 13.92	0.884*
Female	28.28 ± 24.51		10.84 ± 14.12	
Relaps				
Yes	32.12 ± 26.27	0.446	18.67 ± 19.10	0.01*
No	27.06 ± 22.61		8.43 ± 11.76	
Lymphovascular invasion				
Yes	54.86 ± 12.89	0.008	30.34 ± 22.59	0.001*
No	26.23 ± 22.71		9.01 ± 12.22	
Smoking				
Yes	28.93 ± 20.75	0.824	7.95 ± 9.55	0.348*
No	27.65 ± 24.25		11.21 ± 15.14	
Death				
Yes	34.90 ± 24.76	0.012	12.74 ± 16.40	0.14*
No	21.89 ± 20.18		8.19 ± 10.93	

**Figure 2:**
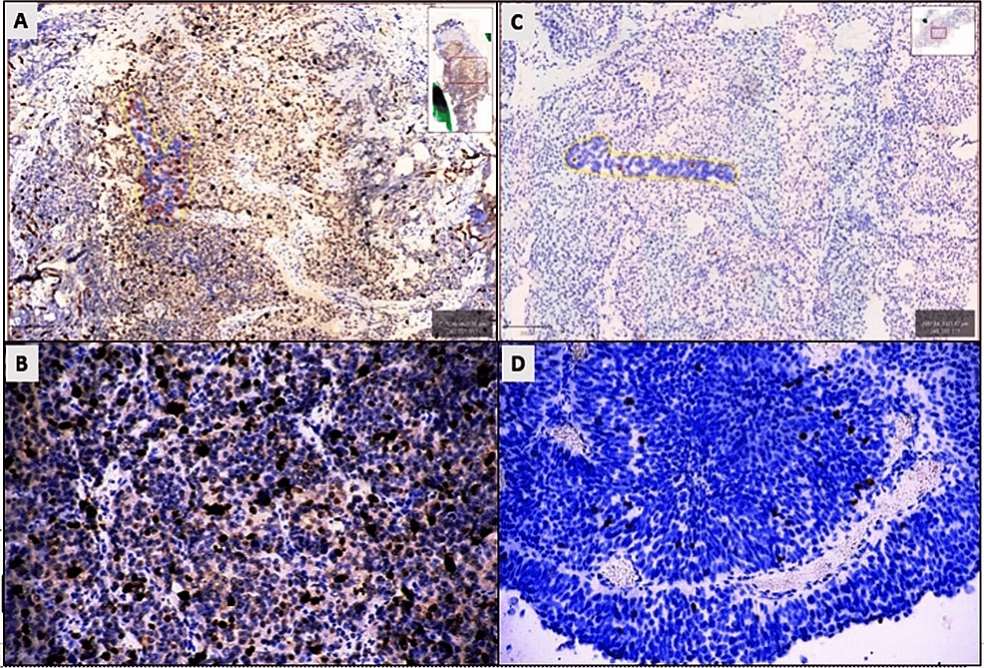
The average pHH3 expression of urothelial carcinoma (A, B) 54.7% nuclear expression in tumoral cells with pHH3 in muscle-invasive HGUC (QuPath 3.0, DAB x200). (C, D) 0.4% nuclear expression in mitotic figures with pHH3 in noninvasive LGUC (QuPath 3.0, DAB x200). LGUC: Low-grade urothelial carcinoma; HGUC: High-grade urothelial carcinoma; pHH3: Phospho-histone 3.

While pHH3 was only expressed in mitotic figures in basal cells of the nontumoral urothelium, it showed nuclear staining in varying amounts in mitotically active tumor cells of urothelial carcinoma. With pHH3, staining was observed in an average of 774.31 ± 1218.5 tumor cells in the urothelial carcinoma hot spot area. The average expression rate of pHH3 in the tumor was determined as 10.32 ± 23.2, ranging from 0.08 to 54.7. In nontumoral urothelium, the average number of cells expressing pHH3 in the hot spot area was 307.4 ± 150.0, and the average pHH3 expression rate was 0.16 ± 1.7, ranging from 0.0 to 6.58 (Figure 2). A statistically significant relationship was detected between the mean percentage of pHH3 expression between the urothelial carcinoma and nontumoral bladder groups (p = 0.018, student's t-test).

When the relationship between pHH3 expression and pT stage was examined, the average pHH3 expression rate was found to be 3.45 ± 4.12 in pTa, 9.35 ± 9.70 in pT1, and 24.54 ± 20.13 in pT2. A statistically significant relationship was detected between pHH3 expression and the pT stage (p < 0.0001, Chi-square test).

The relationship between pHH3 expression and clinicopathological parameters was summarized in Table 2. A statistically significant relationship was detected between pHH3 expression and lymphovascular invasion, histological grade, pT stage, and the presence of recurrence (p < 0.05, Pearson Chi-square test). However, no statistically significant relationship was observed between pHH3 expression and age, gender, death, and smoking (p > 0.05). pHH3 expression positively correlated with lymphovascular invasion (p = 0.018, r = 0.263), pT stage (p < 0.0001, r = 0.588), recurrence (p = 0.010, r = 0.158), and histological grade (p < 0.0001, r = 0.572). No correlation was observed between pHH3 expression and age, gender, smoking, or death (Spearman correlation test).

When the relationship between pHH3 expression and Ki67 expression was examined, the percentage of cells expressing Ki67 was found to be statistically significantly higher than pHH3 in both nontumoral urothelium and urothelial carcinoma tumor cells (p < 0.0001, student's t-test). Additionally, a strong positive correlation was observed between pHH3 expression and Ki67 expression (p < 0.0001, r = 0.665, Spearman correlation test).

**Figure 3:**
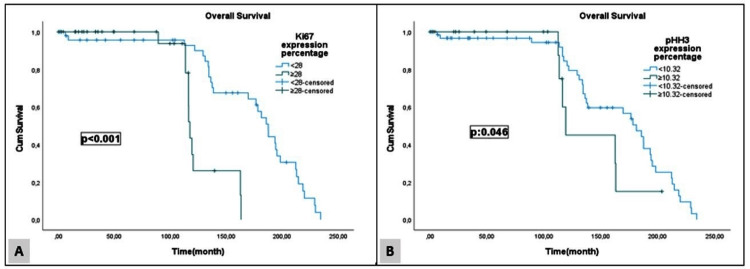
Overall survival Ki67 and pHH3 expression (A) Overall survival curve of Ki67 expression percentage. (B) Overall survival curve of pHH3 expression percentage (Kaplan-Meier analysis). pHH3: Phospho-histone 3.

In the survival analysis of the cases in our study, a statistically significant difference was found between survival and Ki67 and pHH3 expression rates, histological grade, the presence of lymphovascular invasion, and age (p < 0.05). The findings are summarized in Table 3. Figure 3 illustrates the relationship between Ki67 and pHH3 expression rates and survival (Kaplan-Meier analysis).

**Table 3:** Relationship between survival and clinicopathological parameters in urothelial carcinoma cases * Kaplan-Meier analysis, log rank test. LGUC: Low-grade urothelial carcinoma; HGUC: High-grade urothelial carcinoma; pHH3: Phospho-histone 3.

Category	Median Survival (Months) ± SD	p-value
Ki67 expression (%)		
<28	67.95 ± 4.91	<0.0001*
≥28	34.63 ± 4.26	
pHH3 expression (%)		
<10.32	61.78 ± 4.62	0.046*
≥10.32	42.68 ± 8.04	
Age (year)		
<65	77.75 ± 4.99	<0.0001*
≥65	43.58 ± 5.39	
Gender		
Female	50.54 ± 11.22	0.644*
Male	59.78 ± 4.51	
Histologic grade		
HGUC	40.74 ± 4.67	0.003*
LGUC	72.15 ± 5.258	
Lymphovascular invasion		
No	61.90 ± 4.23	<0.0001*
Yes	8.38 ± 3.39	
Pathologic tumor stage		
pTa	73.78 ± 5.26	0.073*
pT1	53.74 ± 6.5	
pT2	24.10 ± 5.54	
Relaps		
No	58.73 ± 4.76	0.90*
Yes	56.68 ± 8.56	
Smoking		
No	61.37 ± 4.79	0.082*
Yes	46.47 ± 7.50	

## Discussion

In this study, the immunohistochemical evaluation of Ki67 and pHH3 expressions in bladder urothelial carcinoma cases was conducted, and these marker expressions were compared with clinicopathological data and prognostic factors.

The Ki-67 index serves as a measure of the rate of cells in the cell division cycle, and this rate is closely associated with the degree and stage of cancer. These characteristics have established Ki67 as a pivotal biomarker for assessing proliferation [19]. In studies across various tumor types, Ki67 has exhibited low expression in benign tumors and high expression in malignant tumors. Furthermore, the expression of this marker has been linked to adverse prognosis in different cancer types.

In a study conducted by Fasching et al. involving breast cancer patients undergoing neoadjuvant therapy, increased Ki67 expression was associated with poor prognosis and reduced survival [20]. In another study concerning papillary thyroid cancer, it was reported that increased Ki67 expression (>20%) was linked to recurrence and lymph node involvement. Moreover, Ki67 expression has been identified as an independent prognostic factor, complementing the TNM classification [21].

Previous research has indicated that Ki-67 expression can serve as an important prognostic marker in bladder cancers [4-6]. In a study by Wang et al., which assessed Ki67, p53, and p63 expressions in bladder cancers, they observed high Ki67 expression in cases with advanced stage, high-grade lymphovascular invasion, and lower survival rates, indicating a poor prognosis [22]. Similarly, Chen et al. stated that increased Ki67 and VEGF expressions in urothelial carcinoma were associated with relapse, progression, and poor prognosis [23]. Furthermore, in a meta-analysis conducted by Tian et al., which included 31 studies and examined Ki67 expressions in 5147 bladder cancer cases, it was emphasized that bladder cancer cases with high Ki-67 expression exhibited lower survival rates and were associated with a more aggressive clinical stage and larger tumor size [6].

In our study, we observed a statistically significant increase in Ki-67 expression rate in tumoral cases compared to the nontumoral control group. These findings suggest that Ki-67 may play a role in the development and progression of bladder cancer. Furthermore, our results indicated that the Ki-67 expression rate was notably higher in advanced-stage tumors, histologically high-grade tumors, and tumors with lymphovascular invasion. All cases with lymphovascular invasion had high-grade muscle-invasive tumors, and these cases exhibited high Ki-67 expression, which was associated with significantly lower survival. Our findings are consistent with the existing literature, where a strong relationship between high Ki-67 expression and factors such as pT stage, histological grade, lymphovascular invasion, and survival has been reported. These results suggest that Ki-67 expression may influence the differentiation, aggressive behavior, and prognosis of bladder cancer.

Although there are studies in the literature evaluating pHH3 expression in various tumor types such as glial tumors, neuroendocrine tumors, breast tumors, and melanomas, there is a limited number of studies investigating pHH3 expression in bladder tumors [16-18].

In our study, we also found a statistically significant increase in pHH3 expression rates in tumoral cases compared to the nontumoral control group, similar to the findings for Ki67. Additionally, pHH3 expression was significantly higher in advanced-stage tumors with lymphovascular invasion, high-grade histological tumors, and cases with a history of recurrence. Furthermore, individuals with high average pHH3 expression rates exhibited lower survival. These findings suggest that pHH3 expression may be a valuable marker for assessing the progression, aggressiveness, and prognosis of bladder cancer, mirroring the trends observed with Ki-67 expression.

There are only a limited number of studies in the literature that have investigated the expressions of Ki67 and pHH3 in bladder carcinomas. In a study by Yin et al., which compared the immunohistochemical expressions of Survivin, Ki67, and PHH3 in superficial urothelial carcinomas, the cut-off value for Ki67 was set at 20%, and for pHH3, the analysis was performed by counting 10-50 expressed cells. They found that noninvasive carcinomas exhibited lower Ki67 and pHH3 expression compared to lamina propria invasive carcinomas. Additionally, high Ki67 and pHH3 values were associated with lower survival and higher recurrence rates [24].

In a study by Mangrud et al., which compared the mitotic activity index (MAI), Ki67, and pHH3 expressions in 193 non-muscle-invasive bladder urothelial carcinomas, it was concluded that increased Ki67 and pHH3 expressions have prognostic value. The study also highlighted the need for further research on proliferation biomarkers in urothelial carcinoma [16].

In our study, when we examined the relationship between pHH3 expression and Ki67 expression, we found that the percentage of cells expressing Ki67 was statistically significantly higher than that of pHH3 in both nontumoral urothelium and urothelial carcinoma cells. This suggests that Ki67 may be a more prominent marker of proliferation in this context.

The limitation of our study was the small sample size.

## Conclusions

In conclusion, our study suggests that Ki67 and pHH3 expressions may be linked to the processes of carcinogenesis, tumor differentiation, aggressiveness, and prognosis of urothelial carcinomas. The assessment of proliferation markers through immunohistochemistry has demonstrated its significance in predicting survival and prognosis across various types of cancers. We believe that our study's findings, which explore the prognostic value of Ki67 and pHH3 as proliferation markers in bladder urothelial carcinomas, should be further validated through additional studies, ideally employing digital imaging analysis methods and larger sample sizes.
